# Developing Mobile- and BIM-Based Integrated Visual Facility Maintenance Management System

**DOI:** 10.1155/2013/124249

**Published:** 2013-10-08

**Authors:** Yu-Cheng Lin, Yu-Chih Su

**Affiliations:** Department of Civil Engineering, National Taipei University of Technology, No. 1 Chung-Hsiao E. Road, Section 3, Taipei 10608, Taiwan

## Abstract

Facility maintenance management (FMM) has become an important topic for research on the operation phase of the construction life cycle. Managing FMM effectively is extremely difficult owing to various factors and environments. One of the difficulties is the performance of 2D graphics when depicting maintenance service. Building information modeling (BIM) uses precise geometry and relevant data to support the maintenance service of facilities depicted in 3D object-oriented CAD. This paper proposes a new and practical methodology with application to FMM using BIM technology. Using BIM technology, this study proposes a BIM-based facility maintenance management (BIMFMM) system for maintenance staff in the operation and maintenance phase. The BIMFMM system is then applied in selected case study of a commercial building project in Taiwan to verify the proposed methodology and demonstrate its effectiveness in FMM practice. Using the BIMFMM system, maintenance staff can access and review 3D BIM models for updating related maintenance records in a digital format. Moreover, this study presents a generic system architecture and its implementation. The combined results demonstrate that a BIMFMM-like system can be an effective visual FMM tool.

## 1. Introduction

Facility maintenance management (FMM) in the operation phase of facility's life cycle has become an important topic for research and academic study. Managing maintenance information about facilities contributes to successful facility management (FM). Managing FMM work effectively can be extremely difficult on the operation phase owing to various types of equipment and facilities. Furthermore, it is inconvenient for maintenance staff to maintain those facilities by relying on paper-based documents. The latest information technology solutions provide improved FMM. Unlike the manufacturing industry, information technology is limited in its use and application in construction [1], and most of the management work is done by human labor, which is inefficient and sometimes error-prone. 

Regarding FMM, maintenance staff usually refers to information such as specifications, checklists, maintenance reports, and maintenance records. Maintenance staff must record maintenance results on hard copies. Consequently, there can be significant gaps in data capture and entry. Such means of communicating information between the facility location and the management office are ineffective and inconvenient. According to the survey findings regarding maintenance work on a commercial building in Taiwan, the primary problems regarding data capture and sharing during the FMM process are as follows: (1) the efficiency and quality are low, especially through document-based media, (2) it is not easy to reference relevant detailed information on facilities, (3) there are data reentry problems, and (4) the use of desktops for operating BIM models cannot be extended to maintenance management service at facility location effectively. However, few suitable platforms exist to assist maintenance staff in using integrated FMM information system from BIM models and sharing maintenance information directly at the facility's location.

**Figure 1:**
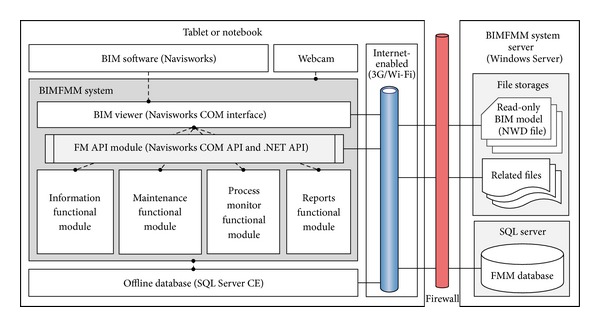
Overview of the BIMFMM system framework.

The performance of FMM can be enhanced by using web technology for information sharing and communication. In this study, the FMM work includes inspection and maintenance works. Building Information Modeling (BIM) uses precise geometry and relevant data to support the maintenance service of facilities depicted in 3D object-oriented CAD. By integrating web and BIM technologies, the effectiveness of FMM work is enhanced and improved (see Figure 1). In order to enhance the effectiveness of FMM work on commercial buildings, this study presents a novel system called BIM-based Facility Maintenance Management (BIMFMM) system for the acquisition and tracking of maintenance information and provides an information sharing platform for maintenance staff using a webcam-enabled notebook or tablet. Integrating the web and BIM technologies, information and data entry mechanisms can help to improve the effectiveness and convenience of information flow in the FMM process. The primary objectives of this study include (1) applying BIM and web technologies to increase the efficiency of collecting maintenance data and information, (2) accessing web technologies directly to link detailed information with BIM models of facilities, and (3) exploring the limitations of the system, addressing problems, and providing suggestions based on the implementation of the pilot case study. The BIMFMM system is applied to a commercial building in Taiwan to verify our proposed methodology and demonstrate the effectiveness of the FMM process. The combined results demonstrate that the BIMMM system can be a useful BIM-based FMM platform by utilizing web and BIM technologies. 

## 2. Related Research Studies

BIM digitally contains precise geometry and relevant data needed to support the design, procurement, fabrication, and construction activities to describe 3D object-oriented CAD [2]. BIM is a digital tool that supports continual updating and sharing of project design information [3]. BIM is the process of generating and managing building data during a building life cycle [4]. BIM technology has the potential to enable fundamental changes in project delivery to support a more integrated, efficient process [5]. Much previous research has examined BIM issues in construction. There are many core benefits, barriers, frameworks, and recommendations on BIM usage cited in previous work on supporting decisions and improving processes throughout the lifecycle of a project [2, 6–14]. Related to the design phase of a project, these topics include parametric modeling, BIM at different levels of development (LOD), identification of design conflicts and analysis, green design, design simulation, cost estimation, and accurate geometric representation of all facilities [2, 7, 8, 15–27]. During the construction phase, these benefits using BIM in construction include less rework, reduction in requests for information and change orders, customer satisfaction through visualization, improved productivity in phasing and scheduling, faster and more effective construction management with easier information exchange, accurate cost estimation, effective supply chain, and visualizing safety analysis [2, 28–35]. During the operation phase, these benefits include control of maintenance management process, integrated life cycle data, rapid and accurate information about update and change activities, and more effective FM with easier information exchange [2, 5, 8, 17, 18, 29, 36–42].

The BIM approach, which is used to retain facility information in a digital format, facilitates easy updates of FMM information in a 3D CAD environment. Although there were many practical applications for using BIM in the maintenance management stage, one of the challenges in broader application of BIM models to FMM is that currently the use of PC desktops limits on-site use of BIM models during the maintenance and inspection process. Another problem for most facilities is that vertical position can be difficult to illustrate clearly based on traditional 2D drawings. With the use of mobile devices executing the BIM models, these BIM models need to be processed in advance and reduced to a smaller file size to be used in mobile devices. This study will explore and make recommendations to solve these problems. In order to assist maintenance staff in obtaining the corresponding BIM model automatically for FMM, this study develops a proposed system to integrate web technology to automatically connect the BIM models. This study enhances FMM service using web technology integrated with the BIM approach. By using the web technology, users can quickly click the corresponding BIM model of a facility and access basic information and maintenance problems while managing FMM information during the operation phase. 

## 3. System Schematic Design

The application of BIM technology in the FMM both inside and outside of the buildings supports maintenance staff in handling FMM via the 3D BIM models. By accessing the mobile device, maintenance staff can obtain the corresponding BIM model of the facility and directly access FMM information about the facility such as instruction manuals, photos, video of operations, maintenance history, and manufacturer information. Furthermore, a 3D BIM model improves upon traditional 2D drawings that can make it difficult to illustrate the vertical location or position of facilities.

The BIMFMM system consists of subsystems for BIM, mobile devices, and a hub center. The mobile devices subsystem is located on the client side, while the BIM models and hub center subsystem are on the server side. Each subsystem is briefly described below.

### 3.1. BIM Modules Subsystem of the BIMFMM System

In this study, BIM is used as an information model in the BIMFMM system. The BIM is applied to capture and store information about the facility, including basic descriptions, parameter-related information, maintenance records, and FMM reports. Autodesk Revit Architecture and Revit MEP were used to create and maintain the BIM model files. Autodesk Navisworks was used to integrate and read the BIM models of facilities. Information integration with the 3D BIM models was achieved using the Autodesk Navisworks API and Microsoft Visual Basic.NET (VB.NET) programming language. The BIMFMM system was developed by integrating the 3D BIM models of facilities and maintenance-related information using Navisworks API programming. Open Database Connectivity (ODBC) was utilized to integrate acquired data from different software programs and all maintenance information, such that BIM files can be exported to an ODBC database for connection with the BIMFMM system. 

### 3.2. Mobile Devices Subsystem of the BIMFMM System

There are two mobile devices used in the BIMFMM system. An Acer Iconia W700P tablet is used as the webcam-enabled tablet hardware. The Acer Iconia W700P tablet runs on Windows 8. An HP Pavilion notebook is used as the webcam-enabled notebook hardware. The HP Pavilion notebook runs the Windows 7 operating system. All data in the tablet and notebook are transmitted between the client and the server sides directly through the web via Wi-Fi or 3G. 

### 3.3. Hub Center Subsystem of the BIMFMM System

The hub center is an information center in the BIMFMM system that enables all participants to log onto a hub center and immediately obtain information required for FMM. Users can access different information and services via a single front-end access point on the Internet. For example, maintenance staff can log onto the hub center and securely access the latest FMM schedule information. FM managers can check maintenance status, results, and various other inspection-related data. All facilities-related pieces of information acquired within the hub center subsystem are recorded in a centralized system database. Maintenance staff can access required information via the hub center subsystem based on their access privileges.

The amount of maintenance information stored will increase over time if all FMM pieces of information are recorded in the BIM model. Because BIM models cover a wealth of building information, central BIM models storage space should be reserved for crucial information, such as spatial information, facility ID and name of facility, facility location, and other critical information. In order to keep the system performance at an acceptable level, the information derived by other applications should be stored in an external location. Therefore, there is one database designed in the BIMFMM system, called the FMM database. The central BIM models stores only basic information (such as position, ID and name of facility, and key parameter information of components). Related maintenance data and information are stored in the FMM database.

The accuracy of the central BIM models will directly affect FMM operations in the BIMFMM system. In order to prevent too many users from simultaneously using central BIM models and in turn affecting the accuracy of the BIM models, the BIM engineer can update central BIM models and export into read-only BIM models (NWD file) directly in the server side. The latest read-only BIM models on the client side automatically re-syncs when read-only BIM models change on the server side. In this framework, all building facility pieces of information from BIM can be saved and updated in the read-only BIM models without accessing the central BIM models directly. Furthermore, central BIM models and read-only BIM model are saved on the server side. Figure 1 shows overview of the BIMFMM system framework.

In the BIMFMM system, major three roles are involved in FMM including BIM engineer, FM manager, and maintenance staff. To ensure that the FMM operation does not affect the maintenance operation of the central BIM models, this study utilizes client-server system architecture. In the BIMFMM system, the read-only BIM model stores all facility basic information and location in the server side. Also, only BIM engineers are allowed to access and edit central BIM models by using BIM software and export into the read-only BIM models directly on the server side. On the client side, the FM manager and maintenance staff reference facility information through the read-only BIM models and edit FMM information through the FMM database in the BIMFMM system. 

**Figure 2:**
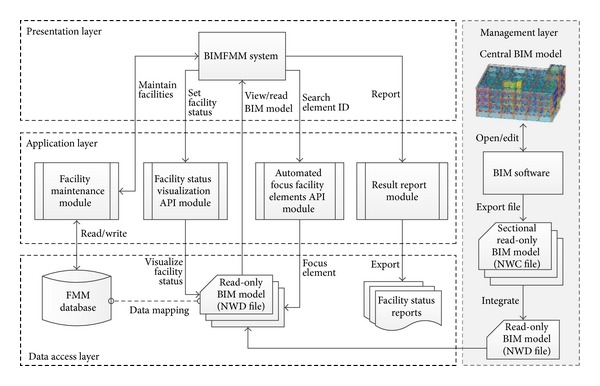
System and module framework of the BIMFMM system.

The BIMFMM system server supports four distinct layers, each with its own responsibilities: management, data access, application, and presentation (see Figure 2). This following section describes the distinct layers in the BIMFMM system.

The management layer provides BIM engineers with tools to edit and manage central BIM models by using BIM software. BIM engineers can create and integrate the read-only BIM models saved in the server through the Internet. 

Regarding the data access layer of the BIMFMM system, the FMM database stores all facilities maintenance records, while the read-only BIM models store complete facility information including facility number, name, and type in the BIM models. The FMM database records detailed maintenance information in accordance with the facility ID. The primary key establishes a relationship between facility ID and the main index. Therefore, information can be used for data association for data mapping to retrieve complete facilities maintenance information based on facility ID between read-only BIM models and FMM database.

The application layer defines various applications for major system and API modules. These applications offer indexing, BIM model data updates and transfers, facility status visualization, and report generation functions. The application layer integrates and uses BIM software to open the BIM models by using developed API modules. Finally, the application layer can automatically acquire data and analyze BIM models based on a request and then send the results back to the client side.

The presentation layer is the main implementation platform of the BIMFMM system. During the FMM process, the FM manager and maintenance staff can use a tablet (client side) and utilities in the BIMFMM system for the FMM operation. The presentation layer displays the location information of BIM model automatically, records maintenance information, illustrates different conditions and status of FMM, queries the history, and exports reports on FMM results. 

## 4. System Development

The BIMFMM system server is based on the Microsoft Windows Server 2008 operating system with an SQL Server 2008 R2 as the database. The BIMFMM system is developed using VB.NET programming, which is easily incorporated with ADO.NET to transact FMM and BIM information with an SQL Server database. The BIMFMM system consists of three different user areas, maintenance staff, FM manager, and BIM engineer areas. Access to the BIMFMM system is password-controlled.

### 4.1. System Functionality Description

This section describes the implementation of each major functionality module in the BIMFMM system.

#### 4.1.1. FMM Information Functional Module

The functional module provides maintenance staff with detailed FMM information on facilities by reviewing 3D BIM models. This module enables all maintenance staff to refer to related FMM information and historical maintenance records for the selected facility quickly and easily in the BIM-based environment. This module allows maintenance staff to refer to basic information and specifications associated with BIM models during the FMM process. This module also has a search function that enables the information to be found and retrieved easily. 

#### 4.1.2. Maintenance Functional Module

Maintenance staff can download up-to-date maintenance records through the BIM models and enter facility maintenance results directly into the BIM models. Additionally, the module can automatically produce the corresponding maintenance forms through the BIM models. Tablets display the checklist for every facility maintenance task. Maintenance staff can record maintenance information such dates, conditions, inspection results, descriptions of problems that have arisen during maintenance, and recommendations. Furthermore, maintenance staff can also check tasks that do not pass the inspection and select relevant tasks from lists in the BIM models. One of the benefits of the module is that maintenance results and records can be transferred between a tablet and the BIMFMM system by real-time synchronization, eliminating the need to enter the same data more than once.

#### 4.1.3. Process Monitor Functional Module

This functional module is designed to enable FM managers to monitor the FMM process. The process monitor module provides an easily accessed and portable environment where maintenance staff can trace and record all maintenance information and statuses through the visualized and colorized BIM model.

#### 4.1.4. Reports Functional Module

Users can easily access the FMM reports functional module to identify needs and analyze FMM results information. Authorized records for interfaces can be extracted and summarized for the final FMM result-related reports. Furthermore, all FMM reports can be extracted using commercially available software such as Microsoft Excel.

### 4.2. System API Modules Description

In order to integrate the system with the BIM models, the following API modules are developed in the BIMFMM system.

#### 4.2.1. Automated Focus Facility Elements API Module

This module allows users easily and quickly to access the related BIM models by entering ID code attached in surface of the facility. When the user enters ID code, this module will automatically identify the facility angle and facility location in the corresponding BIM models automatically for FMM.

#### 4.2.2. Facility Status Visualization API Module

The module provides the visualization functionality for FMM status through a visualized BIM model. Through a systematic FMM analysis of test results, the module displays different colors to illustrate various conditions and FMM status (such as qualified inspection, required repair status, and obsolete facility). Users can access the overall different maintenance conditions and FMM statuses quickly through the visualized BIM model.

There are two subsystems in the BIMFMM system. The first subsystem is the API monitoring subsystem for BIM engineers located on the server side. This subsystem deals with integration services of BIM models in the BIMFMM system. These services include updating facility maintenance information. Another subsystem is the maintenance subsystem located on the client side. This maintenance subsystem is developed for maintenance staff and FM managers to deal with FMM operations in the facility's location, such as clicking the BIM model of the facility, recording FMM, and reporting FMM results. 

### 4.3. System Process Description

There are three processes used in the BIMFMM system including the system initialization process, FMM information monitoring process, and maintenance implementation process.

#### 4.3.1. System Initialization Process

The purpose of the system initialization process is to provide adequate information on FMM operations. The system initialization process includes BIM models initialization and facility information initialization.

*BIM Models Initialization.* The BIM model must provide all pieces of information and related models on a facility as an information requirement for facility maintenance operations. When the BIM models save complete facility information, the BIM engineer needs only to use BIM software (such as Revit) to create BIM models first. After the BIM models creation by Revit can export into many read-only BIM models (NWC file). All exported NWC files will be integrated a single read-only BIM model (NWD file) using Navisworks. Finally, the read-only BIM models (NWD files) can be downloaded on the client side of the BIMFMM system for FMM usage. When central BIM models change, BIM engineers only need to update central BIM models and export to read-only BIM models (NWD files); the BIMFMM system will automatically update read-only BIM model on the client side of the BIMFMM system.

*Facility Information Initialization.* Facility information initialization is an important task in the FMM. After BIM models initialization, FMM staff adds new facility information and electronic documents directly in the system. The information will be saved in the FMM database. Furthermore, the facility information must be linked and associated with BIM models to create the relationship of facility information and BIM models in the MM database.

After the facility information initialization process is completed, FM manager and maintenance staff may utilize BIMFMM system to handle the following FMM information monitoring and maintenance implementation tasks.

#### 4.3.2. FMM Information Monitoring Process

When the system initialization process is completed, FM manager can view and access BIM models (NWD file) in the BIMFMM system and then open the BIMFMM system monitoring API module. When maintenance staff handles the FMM process, FM managers can monitor and refer the newest state of FMM with different color visualization of BIM models through the BIMFMM system.

#### 4.3.3. Maintenance Implementation Process

During the maintenance implementation process, the maintenance list varies according to the maintenance task categories. The design lets maintenance staff work on maintenance operations effectively according to the task categories and maintenance list. Maintenance staff can utilize the Web-enabled Tablet PC to access the BIMFMM system and show all the task categories and maintenance list based on different levels of access. After maintenance staff selects a particular task category, the system shows the history task form for that category. Maintenance staff can view the other task form, edit the unfinished task form, or add a new task form. When maintenance staff selects or adds a task form, the system retrieves facility information from the read-only BIM model based on the task types. Furthermore, a list of all related maintenance and results will be illustrated with the BIM model for FMM work preparation. Maintenance staff can access inspection information and the maintenance status effectively. During the maintenance implementation process, maintenance staff can use the system directly and select the corresponding BIM model of the facility. When the system receives the facility ID, the system automatically displays the facility's basic information and historical maintenance data in the BIM model. Furthermore, the facility's BIM model will be selected, focused, and highlighted using different color. User can obtain basic information on the facility by clicking the BIM model, or selecting from a maintenance list. After selecting the facility through one of the three methods, the maintenance staff can handle maintenance work and record the status and result of maintenance. Finally, all maintenance records and pieces of information are stored in the FMM database.

**Figure 3:**
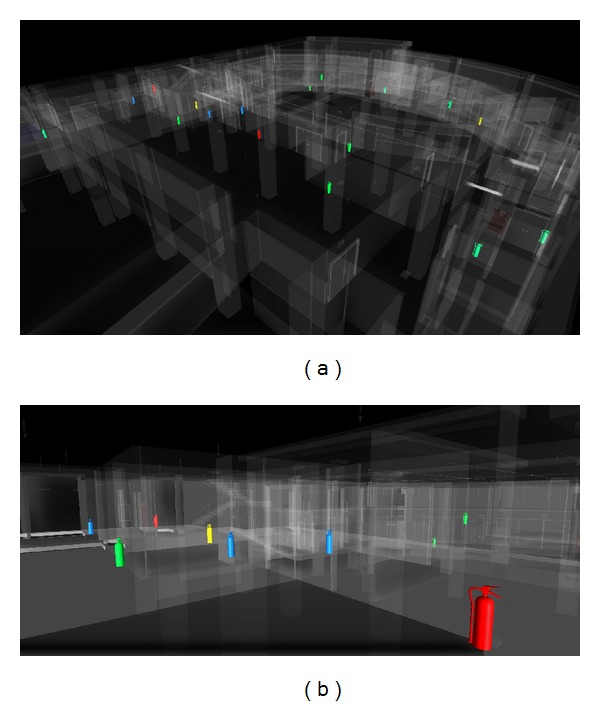
The different maintenance statuses of FMM through visualized and colorized BIM model.

During the process of maintenance operations, the maintenance status also can be enhanced by color visualization in the BIM model through the facility status visualization API module. Through the functionality that visually depicts the status of maintenance list items, the BIMFMM system will get related maintenance status from the maintenance list in the FMM database. Furthermore, the BIM model will visualize different colors based on the each maintenance status in the facilities, and other elements in BIM model will be displayed in translucent white to enhance the visualization effect (see Figure 3).

**Figure 4:**
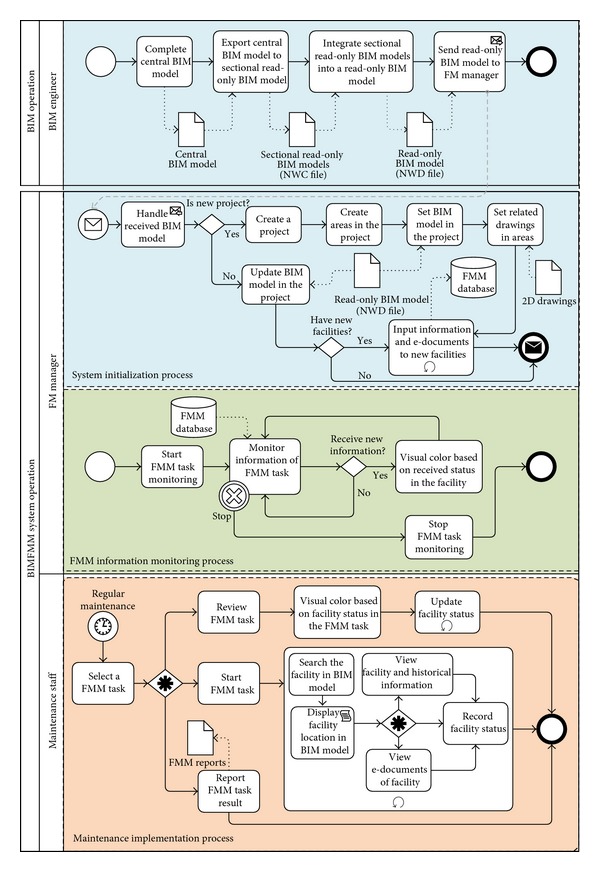
The process diagram used in the BIMFMM system.

**Figure 5:**
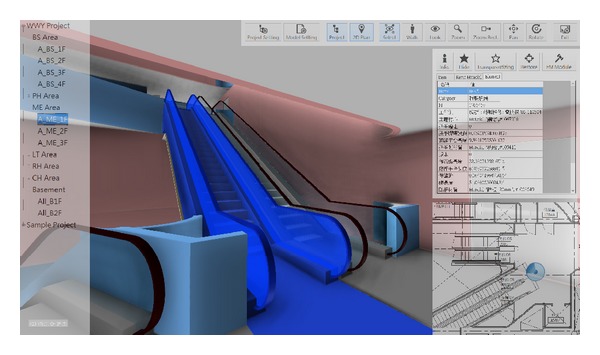
GUI of the BIMFMM system.

Integrated with the above design concept, more complex operating procedures of FMM are simplified and developed in the BIMFMM system. One of the major characteristics of the BIMFMM system is to provide users an easy-to-use visualization for handling FMM work. By clicking the list, each task form will show the list of facilities requiring maintenance, historical maintenance information, and the status and condition of facilities maintenance. By clicking the corresponding BIM model of the facility, the BIM model are linked and illustrated quickly and effectively in facility location. All maintenance results are sent back and saved in the main BIM model. The proposed approach provides a means to update the facility information of the BIM model and FMM information synchronization. Finally, in order to let FMM engineers apply the system easily and effectively, the layout of the system is designed based on FMM engineers' suggestions.  Figure 4 illustrates the system process flowchart used in the BIMFMM system.  Figure 5 shows the graphical user interface (GUI) of the BIMFMM system. 

## 5. System Validation

### 5.1. Pilot Case Study

This study is applied to a building in Taiwan for the case study. This study utilizes a BIMFMM system in the FMM for the building. Existing approaches for tracking and managing FMM work rely on paper-based records. The bulk of FMM work was paper-based and documented by repeated manual entry, although an FMM system was developed for a standalone software application. Therefore, maintenance staff in the FM division utilized the BIMFMM system to enhance FMM work in the pilot case study. 

**Figure 6:**
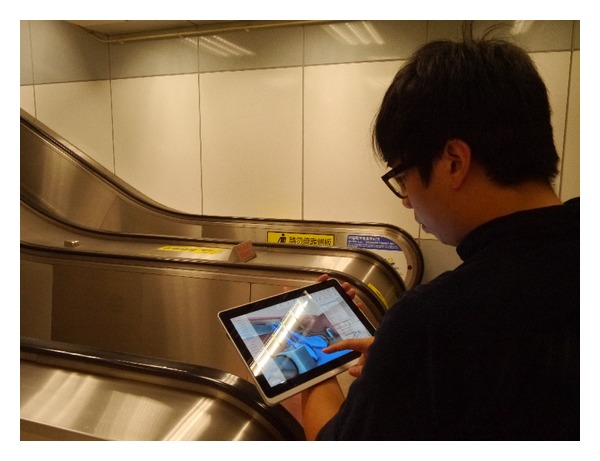
Staff using webcam-enabled tablet for FMM work in the pilot case.

After the critical facilities were selected for FMM work, the unique ID for each facility was entered into the BIMFMM system database for quick search. Before the FMM work began, the maintenance staff could check the facility list from webcam-enabled tablets, refer to the relevant information, and begin preparation work without printing any paper documents. During the FMM process, the maintenance staff selected the relevant BIM model. The BIMFMM system showed the basic information and BIM model of the facility. Maintenance staff could then check further detailed information like maintenance instructions, notifications, and accessories list, all of which are supported by BIMFMM. After the FMM work, maintenance staff entered the results of maintenance, edited the description in the tablet, and provided the updated information to the system. When a facility required repairs, the system also provided the manufacturer's problem information for immediate reference. Finally, the facilities manager and the authorized maintenance staff accessed the updated information simultaneously from their offices.  Figure 6 illustrates maintenance staff using webcam-enabled tablet for FMM work in the pilot case study.

### 5.2. Evaluation and Results

 Overall, the field test results indicate that the application of BIM is an effective tool for FMM in a building. All BIM models survived use in the pilot test over the two-month testing period. Approximately 18 users participated in field trials of the FMM process. The BIMFMM system was installed on the main server in the FM division of the building. 

During the field trials, verification and validation tests were performed to evaluate the system. Verification aims to evaluate whether the system operates correctly according to the design and specification; validation assesses the usefulness of the system. The verification test was carried out by checking whether the BIMFMM system could perform tasks as specified in the system analysis and design. The validation test was undertaken by asking selected case participants to use the system and provide feedback by answering a questionnaire. There were 25 participants involved in the evaluation test. To evaluate system function and the level of satisfaction with the system's capabilities, the users of the system were asked to grade the conditions of system testing, system function, and system capability separately, compared with the typical paper-based FMM approach. Some comments for future improvements to the BIMFMM system were also obtained from the case participants through the user satisfaction survey. Finally, Table 1 shows system evaluation result. 

The 92% of users obtained from user satisfaction survey indicates that the BIMFMM system is quite adaptable to current FMM practices in a building and is attractive to users. The overall result implies that the BIMFMM system is considered to be well designed and could enhance current time-consuming FMM processes. The over 98% satisfaction rate also indicates that the visual BIM model providing FMM support is very helpful. The 98% satisfaction rate for the BIMFMM system directly accessing the BIM model at facility location is also effective and necessary. Also, no additional work was required to complete documentation beyond the data collection process. The advantages and disadvantages of the BIMFMM system identified from the pilot study are identified. 

### 5.3. Limitations and Barriers

The findings of this case study revealed several limitations of the BIMFMM system. The following are inherent problems recognized during the case study.

- It was difficult for new users to operate BIM model in the BIMFMM system. Some maintenance staff was initially unfamiliar with BIM models. It usually takes time to learn how to use BIM models. In the case study, the use of the BIM system initially lengthened the FMM operation over the traditional approach, since users required time to find the corresponding BIM model and fill out the FMM information in the BIMFMM system. After the user is skilled and familiar with BIM model, the time required by the current approach and the proposed system is almost exactly the same in FMM operations.
- Based on the case study, BIM engineers needed to keep and update BIM models during the operation phase. When new equipment or facilities are purchased, BIM engineers must build a new BIM model for future maintenance use. Furthermore, the communications between maintenance staff and BIM engineers are necessary and important during the process. Maintenance staff should tell BIM engineers about any problem regarding the BIM models. The BIM engineers also must notify and discuss with maintenance staff after BIM engineer corrects the BIM models. BIM models require constant maintenance and updates. Another important issue is quality management of BIM models. Although the study proposed that the BIMFMM system help maintenance staff to handle visual facilities maintenance and management work, the advanced management procedures and mechanisms for quality management of BIM models for FMM must be identified and developed in the future. Particularly, the management mechanisms for updating the BIM models should be developed as the next step of BIMFMM system development.
- Although Navisworks provide user with the ability to access a huge integrated amount of BIM models, the integrated BIM models (NWD file) will become larger than original BIM models. Usually, it will take 2 to 5 minutes to download whole BIM models from the server side when applying BIM models in the tablet for FMM work. Therefore, it is necessary to develop appropriate mechanisms to improve the above problem. For example, the BIM models in the client side will be updated and downloaded only from the server side when BIM models changes in the server.

## 6. Conclusions

The BIM approach, which is applied to retain facility information in a digital format, facilitates easy updating of FMM information in a BIM environment. Although there were many practical cases for using BIM during the maintenance management stage, the one of facing problem is that typically BIM models could only be used with PC desktops in an office, which limited their use onsite during facility maintenance. However, use of high-end desktops for operating BIM models could not be used effectively by maintenance staff onsite during the maintenance and inspection process. BIM models need to be processed and transferred via smaller files for use with mobile devices, which are more commonly used onsite. In order to assist maintenance staff with obtaining the corresponding BIM model automatically for FMM, this study develops the BIMFMM system to integrate web technology to automatically connect the BIM models. The BIMFMM system not only improves FMM efficiency but also provides a real-time service platform during the FMM process. In the case study, MM staff used webcam-enabled tablets to seamlessly enhance FMM work at facility locations, owing to the system's searching speed and ability to support related information collection and access during the FMM process. Meanwhile, on the server side, the BIMFMM system offers a hub center to provide the FM division with real time monitoring capacity during the FMM process. Integrated with characteristics of 3D BIM model illustration and BIM parametric design, the BIMFMM system quickly shows the necessary maintenance information using a facility's BIM model based on the selected task type and clearly presents the position and height of the selected facility.

In a case study, the application of the BIMFMM system helped to improve the FMM work of a commercial building in Taiwan. Based on experimental results, this study demonstrated that BIM technology has significant potential to enhance FMM work. The integration of BIM technology with web technology helps FM managers and maintenance staff to effectively track and control the whole FMM process. Compared with current approaches, the combined results demonstrate that a BIMFMM system can be a useful mobile BIM-based FMM platform. Based on the case study finding, BIM models must be updated and corrected constantly. Another important issue is quality management of BIM models. The advanced management procedures and mechanisms for quality management of BIM models for FMM needs to be identified and developed in the future. Doing so will be the next step of BIMFMM system development. Finally, the limitations, facing problems, and suggestions are discussed based on the implementation of case studies in this study. Although there are some challenges indicated above, the proposed system has shown a great potential to be used for FMM in building with the promising results shown in this study. 

## Figures and Tables

**Table 1:** System evaluation result.

System functionality	Mean score
Ease of FMM information sharing	4.4
Reliability	3.9
Applicability to FMM	4.7

Use of system	Mean score

Ease of use	4.4
User interface	4.4
Overall system usefulness	4.4

System capability	Mean score

Reduces unnecessary time	4.3
Ease of finding maintenance information	4.4
Improves maintenance problem tracking	4.0
Enhances visual maintenance management	4.4
Enhances maintenance problems illustration	3.9

Note: the mean score is calculated from respondents' feedback on five scale questionnaire: 1 (strongly disagree), 2, 3, 4, and 5 (strongly agree).
